# Photoreductive Degradation of Fe(III)–Catechol Crosslinked Hydrogels under Visible Light

**DOI:** 10.1002/asia.70725

**Published:** 2026-04-02

**Authors:** Ukhyeon Kim, Beom Jin Kim

**Affiliations:** ^1^ Department of Chemistry University of Ulsan Ulsan Republic of Korea

**Keywords:** drug delivery, gels, photochemistry, redox chemistry, supramolecular chemistry

## Abstract

Hydrogels that degrade in response to external stimuli have attracted interest as dynamic materials for controlled release and biomedical applications. Light is particularly appealing as a non‐invasive trigger, yet most photodegradable hydrogels rely on ultraviolet irradiation, which can induce cellular damage depending on wavelength and dose. Here we present a hydrogel system that undergoes reductive disassembly of Fe(III)–catechol crosslinks under visible light. Incorporation of Fe(III)–citrate into a catechol‐conjugated polymer generated a hydrogel network that could be destabilized upon irradiation. A variety of analyses revealed a clear transition from a gel‐like to a liquid‐like state, confirming that photoreduction of Fe(III) to Fe(II) drives the network degradation. This structural disruption enabled functional release of encapsulated cargo molecules, which was markedly enhanced relative to the controls. Moreover, the release rate could be systematically tuned by light intensity, consistent with photon‐flux‐dependent photoreduction. We envision that the visible‐light‐degradable hydrogels herein have potential applications in spatiotemporally controlled material disassembly across diverse fields.

## Graphical abstract

A hydrogel responsive to visible light was developed via Fe(III)–catechol crosslinking with Fe(III)–citrate. Light irradiation induces photoreduction of Fe(III) to Fe(II), which disassembles the network and causes hydrogel degradation. The resulting structural disruption promotes effective release of encapsulated cargo and offers a platform for stimuli‐responsive soft materials.

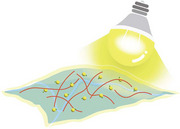

## 1 Introduction

Stimuli‐responsive hydrogels have been extensively investigated as dynamic soft materials, where spatiotemporally controlled degradation is often required for structural disassembly and functional regulation [1, 2, 3]. For this purpose, a variety of external triggers have been developed, including enzymatic cleavage [4], pH variations [5], reducing agents [6], reactive oxygen species (ROS) [7], and thermal stimuli [8]. Among these stimuli, temperature has been one of the most extensively investigated triggers, as thermo‐responsive hydrogels exhibit simple activation mechanisms and reversible sol–gel transitions [9]. However, temperature‐triggered responses are inherently global and therefore lack spatial selectivity, which can limit localized or site‐specific control, particularly in biologically relevant environments [10]. In contrast, light‐based stimuli offer the capability for spatiotemporal control through remote, non‐invasive activation, without perturbing the bulk environment [11, 12]. Owing to these advantages, light has attracted increasing attention as an external trigger for hydrogel degradation. Numerous examples of photodegradable hydrogels have therefore been reported, most of which rely on UV irradiation [13, 14]. However, because UV irradiation can induce cellular damage in a wavelength‐ and dose‐dependent manner, visible‐light‐responsive systems offer a broader biological safety margin [15, 16, 17].

Ferric citrate (Fe(III)–citrate) is known to exhibit photoreduction under natural light, whereby Fe(III) are converted into Fe(II) [18]. This photoreduction plays a central role in regulating iron bioavailability and transport in plants [19], and has been directly linked to iron metabolism in leaves and xylem sap [20]. Accordingly, Fe(III)–citrate is not just a simple metal–organic complex but also a functional unit capable of participating in light‐induced redox processes in natural systems. These observations further suggest that Fe(III)–citrate could also mediate light‐driven reduction processes in artificial environments.

Catechol (Cat) groups coordinate strongly with Fe(III), generating reversible and dynamic crosslinks within hydrogel networks [21, 22]. Fe(III)–Cat interactions have been widely explored as dynamic coordination motifs responsive to external stimuli such as pH variation and redox conditions, enabling reversible modulation of network mechanics [23, 24, 25]. Accordingly, Fe(III)–Cat hydrogels have been applied to mussel‐inspired adhesives, self‐healing materials, and drug delivery systems [26, 27, 28]. In most prior studies, however, these external stimuli primarily induced reversible changes in coordination equilibrium or network stiffness rather than irreversible degradation of the hydrogel structure. Direct light‐triggered disruption of Fe(III)–Cat coordination crosslinks via photoreduction, leading to macroscopic hydrogel disassembly, has not been systematically demonstrated. Moreover, previously reported visible‐light‐responsive hydrogels have predominantly relied on photocleavable covalent linkers or photo‐induced softening of polymer networks [29]. In contrast, coordination‐driven redox processes enabling macroscopic hydrogel disassembly have remained largely unexplored. Leveraging metal–ligand photoreduction to trigger network collapse therefore represents a mechanically distinct strategy compared to conventional photocleavage‐based systems.

**FIGURE 1:**
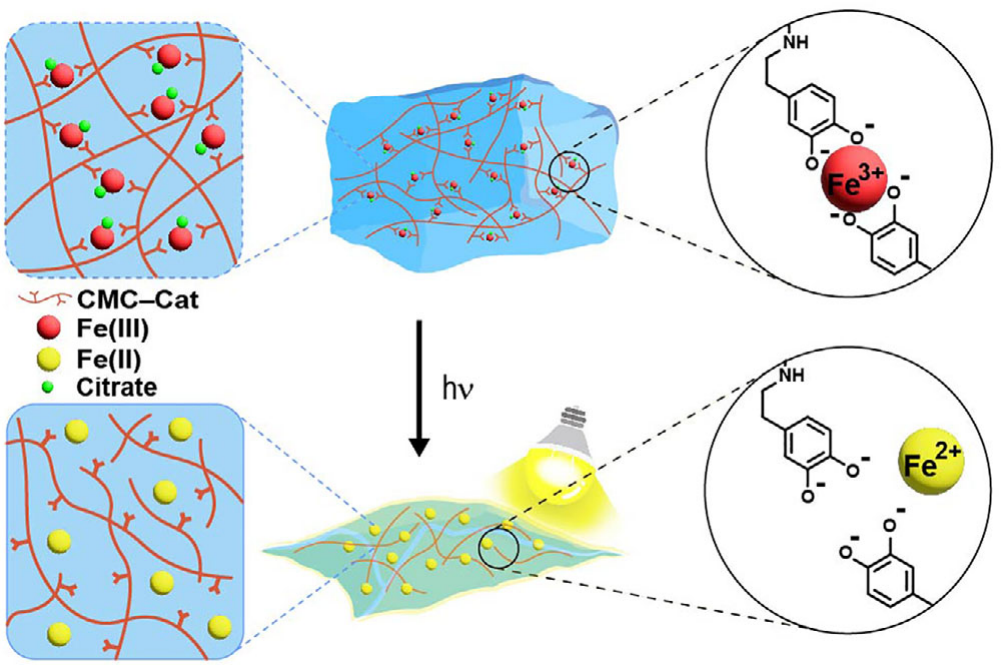
Schematic representation of photoreductive degradation of Fe(III)–catechol (Fe(III)–Cat) crosslinked hydrogel under visible light.

Herein, we introduce a visible‐light‐responsive hydrogel system inspired by the naturally occurring photoreduction of Fe(III)–citrate (Figure 1). The hydrogel was synthesized by incorporating Fe(III)–citrate into a Cat‐conjugated polymer solution, thereby forming Fe(III)–Cat crosslinks that established the hydrogel network. Upon visible‐light irradiation, Fe(III) functioning as the crosslinker was reduced to Fe(II), which triggered disassembly of the hydrogel network and ultimately led to its degradation. In particular, we sought to distinguish between the intrinsic structural stability of the assembled hydrogel network and externally triggered disassembly arising from Fe(III)–Cat photoreduction. As a demonstration, rhodamine B encapsulated within the hydrogel was effectively released upon visible light exposure as a consequence of the network disruption. This visible‐light‐induced reductive degradation mechanism establishes a basis for designing degradable hydrogels relevant to controlled drug release, tissue engineering and regenerative medicine, and stimuli‐responsive biomaterials.

## 2 Results and Discussion

**FIGURE 2:**
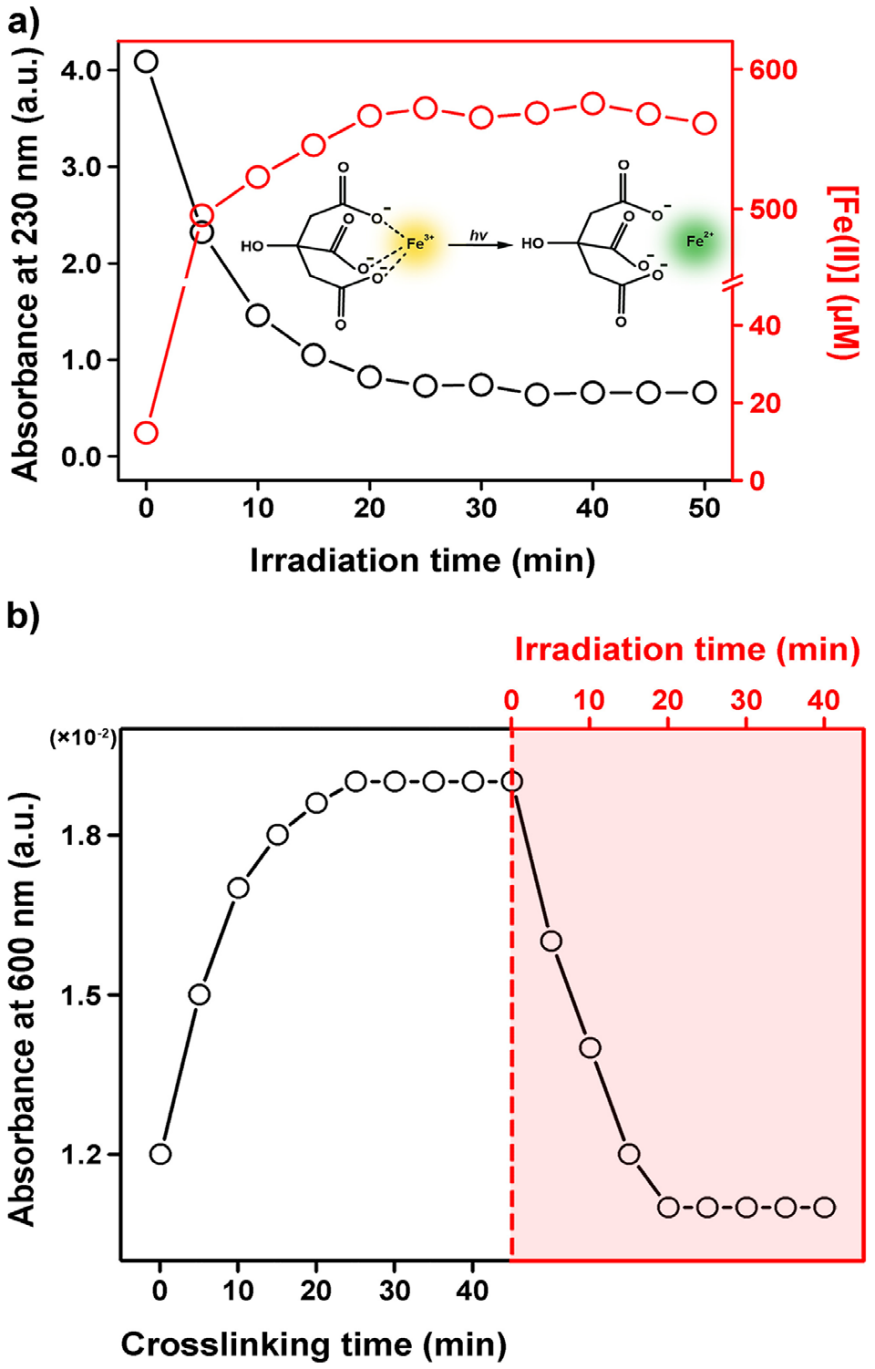
Photoreduction of Fe(III)–citrate and disassembly of Fe(III)–Cat crosslinks under visible light. a) Change in the characteristic Fe(III)–citrate shoulder peak at 230 nm (black) with Fe(II) concentration (red) during irradiation. b) Increase in the Fe(III)–Cat ligand‐to‐metal charge transfer (LMCT) absorbance at 600 nm during the crosslinking, followed by its decrease under irradiation.

The Fe(III)–citrate complex exhibits a characteristic shoulder peak in its ultraviolet–visible (UV–vis) absorption spectrum near 230 nm, which is typically attributed to ligand‐to‐metal charge transfer (LMCT) transitions in related Fe(III)–carboxylate complexes (Figure S1) [30]. Therefore, we monitored the change in this absorption as a function of visible‐light irradiation time (Figure 2a). The results showed that the absorbance at 230 nm gradually decreased with increasing irradiation time, indicating that the Fe(III)–citrate complex undergoes photoreduction upon visible‐light exposure in a time‐dependent manner. This light‐induced transformation of the Fe(III)–citrate complex was further supported by Fourier transform infrared (FT‐IR) analysis (Figure S2). Specifically, a band at ∼1725 cm^−1^, associated with the carbonyl stretching vibration of carboxylic acid groups in citrate species, was markedly attenuated after visible‐light irradiation, indicating disruption of the citrate coordination environment [31]. Concurrently, an increase in the absorption band at ∼2340 cm^−1^, corresponding to the asymmetric stretching of CO_2_, was observed. The evolution of this band suggests partial decarboxylation of citrate accompanying the photoreduction process [32].

Since Fe(III) was reduced to Fe(II) during this process, the concentration of reduced Fe(II) was quantitatively analyzed over irradiation time. A standard calibration curve was first established using aqueous Fe(II) solutions of known concentrations, where Fe(II) formed a coordination complex with 1,10‐phenanthroline (phen), producing a characteristic absorption peak at 510 nm (Figure S3) [33]. As the reduced Fe(II) generated during irradiation was also chelatable with phen, the resulting absorption at 510 nm was compared with the calibration curve to determine the reduced Fe(II) concentration. The analysis revealed that the concentration of the reduced Fe(II) increased progressively with irradiation time and reached saturation of approximately 560 µM after 20 min, beyond which no further reduction was observed. These results demonstrate that Fe(III) coordinated with citrate is effectively reduced upon visible‐light irradiation.

Cat exhibits exceptionally high affinity toward Fe(III) (e.g., log *K*_S_ ≈ 37–40; *K*_S_ is the equilibrium constant for complex formation) [23], and such Fe(III)–Cat coordination has been employed in Cat‐conjugated polymer systems for applications including multilayer antibacterial coatings based on Cat–Fe(III)–Cat linkages as well as Fe(III)‐mediated stabilization of polydopamine coatings [34, 35]. Accordingly, dopamine was conjugated to carboxymethyl cellulose (CMC) to introduce Fe(III)‐binding Cat groups, yielding Cat‐functionalized CMC (CMC–Cat) (Figure S4). To verify successful Cat conjugation to CMC, we first measured the UV–vis absorption spectrum of the purified CMC–Cat. Free Cat in aqueous solution exhibits a characteristic absorption band near 275 nm attributable to a π–π* transition [36, 37], and a similar band was observed for CMC–Cat, indicating the presence of Cat moieties in the polymer (Figure S5a). Further structural confirmation was obtained by FT‐IR and ^1^H NMR analyses. In the FT‐IR spectrum, compared with pristine CMC, CMC–Cat exhibited new absorption bands at ∼1592 cm^−1^, which can be attributed to amide II vibrations (N–H bending coupled with C–N stretching), while the band at ∼1065 cm^−1^ corresponds to C–N stretching vibrations (Figure S5b) [38]. These features are consistent with amide bond formation between the amine group of dopamine and the carboxyl groups of CMC. In addition, ^1^H NMR analysis revealed the appearance of characteristic aromatic proton signals derived from dopamine, which were absent in pristine CMC (Figure S5c). The emergence of these dopamine‐specific peaks provides direct molecular evidence for successful catechol grafting onto the CMC backbone. Next, Fe(III)–citrate was added to an aqueous solution of CMC–Cat, and Fe(III)–Cat crosslinking was allowed to proceed for 45 min (Figure 2b). Formation of crosslinks was verified by an increase in absorbance at 600 nm arising from the LMCT band [39], and subsequent visible‐light irradiation led to a gradual decrease of this absorbance over time, evidencing disassembly of the Fe(III)–Cat crosslinks via photoreduction. Taken together, these results show that the simple incorporation of Fe(III)–citrate to CMC–Cat is sufficient to induce Fe(III)–Cat crosslinking and that the crosslinks can be disrupted by visible‐light‐induced reduction of Fe(III) to Fe(II).

We next sought to form a hydrogel network by increasing the concentrations of CMC–Cat and Fe(III)–citrate, given that their reaction had been confirmed to generate Fe(III)–Cat crosslinks. Specifically, an aqueous solution containing 4.5% (w/w) CMC–Cat and 300 mM Fe(III)–citrate was vortexed, and the gelation process was monitored over time. Complete hydrogel formation was observed after approximately 40 min (Figure S6a). Rheological strain sweep measurements performed at selected time points after mixing revealed that the formation of an elastic‐dominant network, in which the storage modulus (*G*′) exceeded the loss modulus (*G*″) (i.e., *G*′ > *G*″), within 10 min. The *G*′ value progressively increased and reached a plateau at approximately 40 min (Figure S6b), indicating completion of network maturation and stabilization of the Fe(III)–Cat coordination crosslinks. To evaluate the effect of Fe(III) concentration on hydrogel mechanical strength, rheological analyses under strain sweep conditions were conducted on hydrogels prepared by varying the Fe(III)–citrate concentration while maintaining the CMC–Cat concentration at 4.5% (w/w) (Figure S7). As the Fe(III)–citrate concentration increased, *G*′ in the linear viscoelastic regime increased accordingly, indicating enhanced network strength due to increased Fe(III)–Cat crosslink density. This trend is consistent with coordination‐mediated network formation, whereby increasing Fe(III) concentration enhances network connectivity and crosslink density until a mechanically stable regime is achieved. In particular, a pronounced increase in both *G*′ and *G*″ was observed when the Fe(III)–citrate concentration reached 300 mM, whereas hydrogels prepared at lower concentrations exhibited substantially weaker mechanical properties. Based on these results, 300 mM Fe(III)–citrate was selected as an optimized condition balancing mechanical stability and efficient light‐triggered degradation. The mechanical robustness of the optimized hydrogel under large deformation was subsequently assessed through cyclic strain sweep experiments, in which the applied strain alternated between low (0.1%) and high (300%) amplitudes. A reversible decrease and recovery of the viscoelastic moduli were observed during successive strain cycles, indicating that the Fe(III)–Cat coordination network can withstand and recover from transient structural disruption (Figure S8a). Such reversible behavior suggests that the hydrogel possesses mechanical adaptability under high strain, a feature that is advantageous for potential injectability (Figure S8b).

**FIGURE 3:**
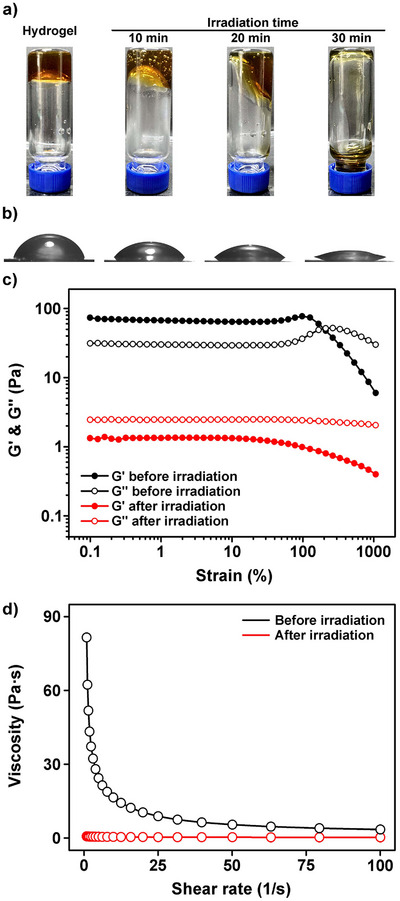
Visible‐light‐induced degradation of Fe(III)–Cat crosslinked hydrogel. a) Photographs of hydrogel before and after irradiation at different times. b) Contact angle measurements during irradiation. c) Storage (*G*′) and loss (*G*″) moduli before and after irradiation. d) Viscosity profiles before and after irradiation.

To investigate whether the resulting hydrogel undergoes photoreduction‐induced disassembly of Fe(III)–Cat crosslinks, as observed at the molecular level under dilute conditions, the hydrogel was irradiated with visible light while monitoring its state (Figure 3a). The hydrogel showed gradual changes in its physical properties with irradiation time, and after 30 min, the hydrogel network was disrupted, yielding the polymer solution‐like phase. This transition was further confirmed by contact angle measurements (Supporting Movie). A 10 µL volume of the hydrogel was formed on a gold wafer, and the apparent contact angle was monitored during irradiation as a quantitative indicator of spreading behavior associated with hydrogel disassembly (Figure 3b). The contact angle of the hydrogel, initially 68.20 ± 0.12° before irradiation, gradually decreased to 58.85 ± 0.45° after 10 min, and 34.88 ± 0.50° after 20 min. After 30 min of irradiation, the contact angle reached 16.22 ± 0.39°, reflecting pronounced spreading arising from disruption of the self‐supporting hydrogel network and formation of a liquid‐like phase.

The UV–vis absorption spectrum of the intact Fe(III)–Cat crosslinked hydrogel was obtained prior to irradiation (Figure S9). The hydrogel exhibits broad absorption across the visible region, demonstrating that the coordination network can effectively absorb the broadband visible light employed and thereby undergo photoreduction‐induced disassembly. The macroscopic disassembly of the hydrogel was further corroborated at the molecular coordination level by monitoring the LMCT band of the Fe(III)–Cat crosslinked hydrogel during visible‐light irradiation (Figure S10). The absorbance at 600 nm gradually decreased with irradiation time, indicating progressive disruption of Fe(III)–Cat crosslinks within the hydrogel network. This time‐dependent decrease reached a near‐plateau within 60 min, suggesting substantial coordination bond disruption. Based on this observation, an irradiation time of 60 min was selected as a representative condition to ensure complete hydrogel disassembly in subsequent experiments.

Atomic force microscopy (AFM) imaging was also conducted to examine nanoscale structural evolution of the hydrogel network before and after visible‐light irradiation (Figure S11). The intact hydrogel displayed a continuous and interconnected network morphology with an average surface roughness (*R*_a_) of 9.34 ± 1.80 nm. Upon irradiation, the network‐like features became fragmented and flattened, and *R*_a_ markedly decreased to 0.84 ± 0.20 nm. This substantial reduction in *R*_a_ quantitatively confirms nanoscale structural collapse of the coordination‐crosslinked network.

To quantitatively evaluate the mechanical changes of the hydrogel associated with photoreduction‐induced disassembly, we performed rheological analyses. Strain sweep measurements of *G*′ and *G*″ revealed that, prior to irradiation, *G*′ was markedly higher than *G*″, characteristic of solid‐like hydrogel behavior (Figure 3c) [11]. After irradiation, however, *G*′ significantly decreased and became lower than *G*″, demonstrating a transition from solid‐like to viscous‐dominant behavior, consistent with the disruption of the hydrogel network [40]. The residual *G*′ observed after irradiation can be attributed to the intrinsic viscoelasticity of the CMC–Cat polymer solution remaining after substantial disruption of the crosslinked network. In addition, frequency sweep rheological measurements were performed to examine the frequency‐dependent viscoelastic behavior of the hydrogel (Figure S12). Prior to irradiation, *G*′ remained higher than *G*″ over the entire frequency range tested, indicating a stable elastic‐dominant network. After irradiation, both *G*′ and *G*″ significantly decreased and exhibited enhanced frequency dependence with *G*″ becoming higher than *G*′ across the measured frequency range, reflecting substantial weakening and dynamic disruption of the Fe(III)–Cat crosslinked hydrogel network. To further verify this transition, we also measured the viscosity (Figure 3d). Before irradiation, the hydrogel exhibited substantially higher viscosity at low shear rates, which decreased sharply with increasing shear rate, reflecting typical shear‐thinning behavior [41]. In contrast, after irradiation, the viscosity was markedly reduced and became nearly independent of shear rate, showing Newtonian‐like fluid characteristics. These findings are consistent with the contact angle and visual observations, and together confirm that photoreduction of Fe(III)–Cat crosslinks results in complete network disruption and transformation of the hydrogel into the CMC–Cat polymer solution‐like state.

To evaluate long‐term thermal stability under physiological conditions, the hydrogel was maintained at 37°C in the dark for up to 28 days (Figure S13). Periodic strain sweep rheological measurements revealed that *G*′ remained higher than *G*″ throughout the incubation period, confirming preservation of the elastic network structure without spontaneous degradation. Notably, even after 28 days of incubation, the hydrogel retained its photo‐responsiveness and underwent rapid disassembly upon visible‐light irradiation. These findings indicate that the hydrogel network remains structurally stable under physiological conditions in the absence of light, suggesting that disassembly is not driven by spontaneous equilibrium processes but is instead externally triggered by photochemical activation.

**FIGURE 4:**
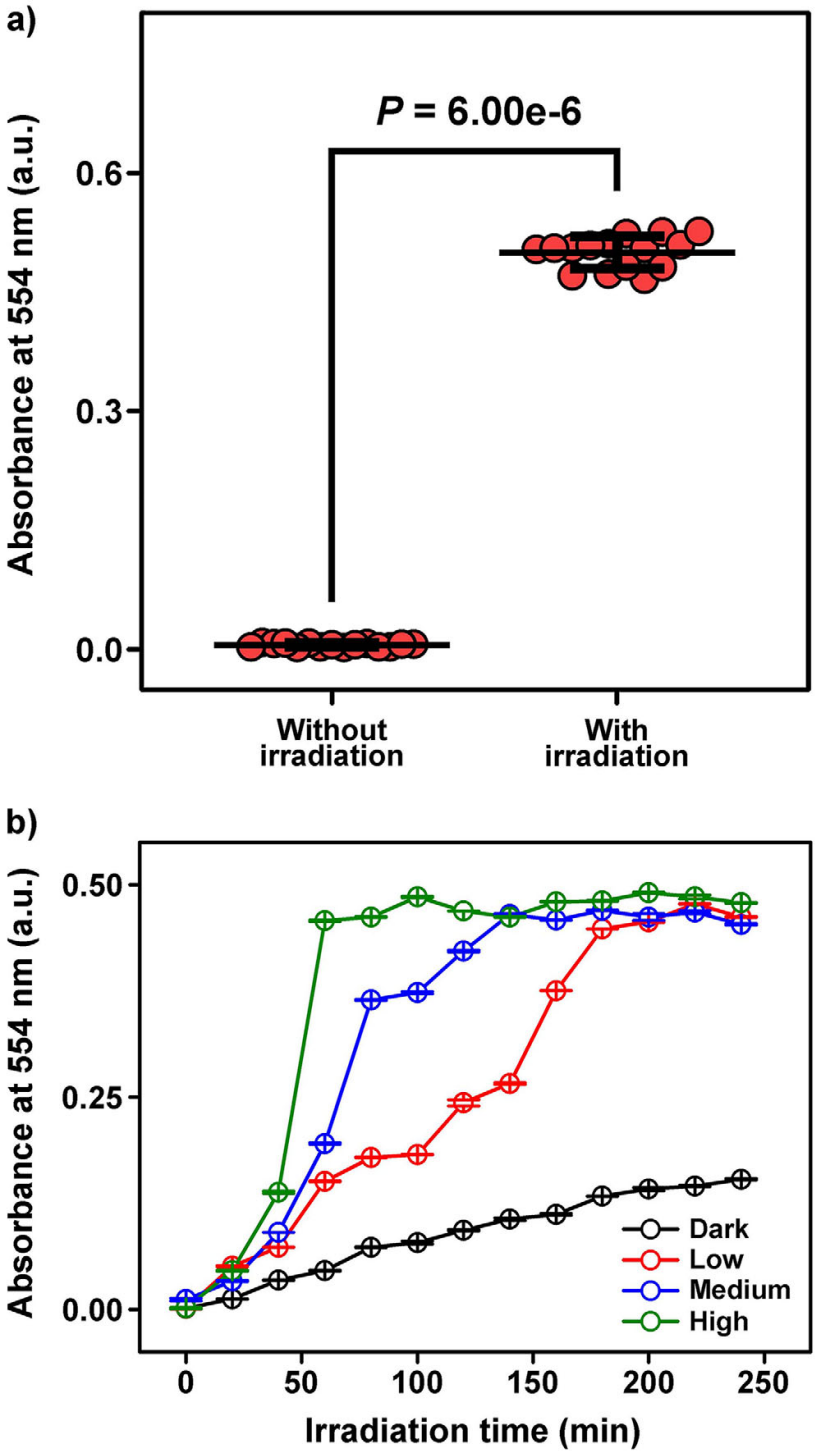
Visible‐light‐triggered cargo release from Fe(III)–Cat crosslinked hydrogel. a) Absorbance of released rhodamine B at 554 nm under without irradiation and with irradiation conditions. Individual data points represent independent experiments (*n* = 15). Statistical significance was evaluated using Student's *t*‐test. b) Time‐dependent release profiles of rhodamine B under dark conditions, and low (71.9 mW/cm^2^), medium (108.2 mW/cm^2^), and high (141.3 mW/cm^2^) light intensity.

Having established that Fe(III)–Cat crosslinks undergo photoreduction‐induced disruption under visible light, we next examined whether this mechanism could effectively mediate the release of cargo molecules encapsulated within the hydrogel. As a model cargo, rhodamine B was encapsulated in the hydrogel and subsequently subjected to visible‐light irradiation (Figure 4a). After irradiation, degradation of the hydrogel was accompanied by a pronounced release of rhodamine B. The extent of release was quantitatively evaluated by monitoring the absorbance at 554 nm using UV–vis spectroscopy [42]. Compared with the baseline release observed under non‐irradiated conditions, visible‐light‐induced hydrogel degradation resulted in about 85‐fold increase in the absorbance, demonstrating that the network disruption triggered by visible light effectively mediates cargo release. The degraded hydrogel prepared without rhodamine B exhibited negligible absorbance at 554 nm after visible‐light irradiation, indicating that degradation products of the hydrogel do not interfere with rhodamine B quantification (Figure S14).

We further investigated the correlation between the photoreduction process and cargo release behavior by modulating the light intensity applied to the hydrogel. While the preceding experiments were performed at high light intensity, the hydrogel was subsequently subjected to three different light powers—high, medium, and low—under otherwise identical conditions (Table S1). Whereas a hydrogel maintained under dark conditions exhibited only minimal diffusion‐driven release, visible‐light irradiation led to markedly accelerated release of rhodamine B (Figure 4b). This effect can be attributed to the increased photon flux associated with higher light intensity at high light power, which promoted faster reduction of Fe(III) and consequently accelerated disassembly of the Fe(III)–Cat crosslinks, leading to rapid degradation of the hydrogel network. In contrast, under low light power, the photoreduction process proceeds more gradually, resulting in slower network disassembly and a correspondingly gradual release of rhodamine B. The systematic increase in the apparent zero‐order release rate constant (*k*_0_), from 2.18 under low light power to 3.56 at medium light power and 7.33 at high light power, further indicates that the release behavior is kinetically coupled to the rate of photoreduction occurring within the hydrogel network (Figure S15). These findings demonstrate that the visible‐light intensity serves as a critical parameter governing the process of hydrogel photoreductive degradation and the release rate of encapsulated cargo molecules.

## 3 Conclusion

We have reported a hydrogel system that responds to visible light through reductive disassembly of Fe(III)–Cat crosslinks. Inspired by the photoreduction of Fe(III)–citrate in natural systems, we showed that Fe(III) crosslinkers are converted to Fe(II) under visible‐light irradiation, resulting in degradation of the hydrogel network. The transition, confirmed by rheological and viscosity analyses together with contact angle studies, indicated a shift from a gel‐like material to a liquid‐like state. This loss of structural integrity was reflected in function: encapsulated cargo molecules were released much more efficiently upon light exposure, exceeding non‐irradiated controls by about 85‐fold. Moreover, by varying the applied light intensity, we observed that the cargo release rate increased with photon flux, suggesting that higher photon flux accelerates photoreduction and thereby promotes the network disassembly. Our findings provide a design framework for hydrogels that integrate dynamic metal–ligand coordination with visible‐light photochemistry. We anticipate that this strategy offers opportunities for spatiotemporally controlled material decomposition and maybe harnessed for precision drug delivery, tissue repair and regeneration, and advanced smart biomaterials with adaptive functions.

## 4 Experimental Section

### 4.1 Materials

All chemical reagents and solvents were used without additional purification. Dopamine hydrochloride (99%, Thermo Fisher Scientific), sodium carboxymethyl cellulose (CMC, Mw ∼90,000, Sigma‐Aldrich), sodium hydroxide (NaOH, 98%, Samchun), 2‐morpholin‐4‐ylethanesulfonic acid (MES, ≥99%, Sigma‐Aldrich), 1‐(3‐dimethylaminopropyl)‐3‐ethylcarbodiimide hydrochloride (EDC·HCl, >98%, TCI), *N*‐hydroxysuccinimide (NHS, 98%, Daejung), 1‐hydroxybenzotriazole hydrate (HOBt, >98%, Daejung), ethylenediaminetetraacetic acid disodium salt dihydrate (EDTA, 99%–101%, Sigma‐Aldrich), sodium bicarbonate (NaHCO_3_, 99‐100.5%, Samchun), ferric citrate (Fe(III)–citrate, Sigma‐Aldrich), rhodamine B (Daejung), iron(II) chloride tetrahydrate (FeCl_2_ ∙ 4H_2_O, Sigma‐Aldrich), hydroxylamine hydrochloride (NH_2_OH·HCl, Sigma‐Aldrich), 1,10‐phenanthroline monohydrate (phen, >98%, TCI), and sodium acetate (anhydrous, ≥98.5%, Samchun) were used as received. White light source (A160WE, Kessil), dialysis tubing (MWCO: 12–14 kD, Thermo Fisher Scientific), and deionized (DI) water (18.3 MΩ cm) from Milli‐Q Direct 8/16 Water Purification System (Merck Millipore) were used.

### 4.2 Measurement of Fe(III)–Citrate Photoreduction

#### 4.2.1 Time‐Resolved UV–Vis Absorption Analysis of Fe(III)–Citrate during Photoreduction

Fe(III)–citrate solutions were prepared by dissolving Fe(III)–citrate in MES buffer (100 mM, pH 6.0) to a final concentration of 750 µM. Each sample was irradiated with visible light for up to 50 min at 5‐min intervals, after which it was immediately analyzed by UV–vis spectroscopy to obtain its absorption spectrum. Since Fe(III)–citrate shows a characteristic shoulder peak at 230 nm, the decrease in absorbance at this wavelength was monitored with irradiation time to evaluate the extent of Fe(III)–citrate photoreduction.

#### 4.2.2 Quantification of Fe(II) Generated During Photoreduction using a Calibration Curve

To establish a standard calibration curve, FeCl_2_ was dissolved in MES buffer (100 mM, pH 6.0) to prepare a series of seven FeCl_2_ solutions with known concentrations (1.5 mL each). Subsequently, 10% (w/w) NH_2_OH, 0.15% (w/w) phen, and 1 M sodium acetate were sequentially added to each solution, followed by incubation for 30 min. After incubation, the absorption spectra were obtained by UV–vis spectroscopy, and the absorbance at 510 nm was used to establish the calibration curve. For quantification of Fe(II) generated during photoreduction, a Fe(III)–citrate solution at a concentration of 750 µM was prepared in MES buffer (100 mM, pH 6.0) and irradiated with visible light for up to 50 min at 5‐min intervals. After irradiation, the samples were processed following the same procedure used for establishing the calibration curve. The absorbance at 510 nm was then measured, and the Fe(II) concentration was determined by comparison with the standard calibration curve.

### 4.3 Synthesis of CMC–Cat

CMC–Cat was synthesized via amide coupling between the carboxyl groups of CMC and the amine group of dopamine using EDC/NHS/HOBt as coupling reagents. CMC (0.4 g) was dissolved in 40 mL of MES buffer (100 mM, pH 4.5) and stirred for 2 h. Subsequently, EDC, NHS, and HOBt (236.4, 28.4, and 189 mg, respectively) were added to the CMC solution and stirred for 1 h under a nitrogen atmosphere. Dopamine hydrochloride (468 mg) was dissolved in 5 mL of MES buffer (100 mM, pH 4.5), stirred for 30 min under nitrogen, and then injected into the CMC solution using a syringe, after which resulting mixture was stirred for 24 h. After the reaction, the product was purified by dialysis (MWCO: 12–14 kDa) against DI water, freeze‐dried, and stored in a refrigerator until use. The successful synthesis of CMC–Cat was confirmed by obtaining the UV–vis absorption spectrum of the aqueous CMC–Cat solution and verifying the presence of a peak at 280 nm. The degree of Cat modification of CMC was determined by UV–vis spectroscopic analysis following a previously reported method [38]. After removal of unreacted dopamine by dialysis, the amount of conjugated Cat was quantified using a calibration curve of dopamine at 280 nm (Figure S16). The Cat grafting density was calculated based on the molar ratio of conjugated Cat to the repeating monomer units of CMC, yielding a degree of substitution of 4.89 mol%.

### 4.4 Analysis of Fe(III)–Cat Crosslinks

The formation of Fe(III)–Cat crosslinks and their dissociation induced by the photoreduction of Fe(III) were monitored by measuring absorbance at 600 nm. CMC–Cat and Fe(III)–citrate were dissolved in MES buffer (100 mM, pH 6.0) at concentrations of 0.1% (w/w) and 6 mM, respectively, to induce Fe(III)–Cat crosslinking. After 45 min of crosslinking, the samples were further irradiated with visible light for 40 min to induce photoreduction of Fe(III). Throughout the crosslinking and dissociation processes, the absorbance at 600 nm was obtained at 5‐min intervals.

### 4.5 Formation and Photodegradation of Hydrogel from CMC–Cat and Fe(III)–Citrate

#### 4.5.1 Formation of Hydrogel

CMC–Cat and Fe(III)–citrate were dissolved in MES buffer (100 mM, pH 6.0) to final concentrations of 4.5% (w/w) and 300 mM, respectively, and mixed by vortexing for 20 s. The mixture was incubated for 1 h to allow sufficient Fe(III)–Cat crosslinking. For rhodamine B encapsulation, the hydrogel was formed using the same procedure, while including rhodamine B in the MES buffer at a final concentration of 1 mM.

#### 4.5.2 Photodegradation of Hydrogel

The formed hydrogels were placed in an aluminum foil‐shielded box and exposed to visible light from the top to induce the degradation. During the degradation process, photographs were taken at 10‐min intervals to monitor morphological changes of the hydrogel. The release of rhodamine B from the hydrogels was investigated with and without visible‐light irradiation. To each rhodamine B‐encapsulated hydrogel, 2 mL of MES buffer was added, and degradation was induced by irradiation for 1 h under the same conditions. As a control, a parallel hydrogel was incubated for 1 h in the absence of light. The supernatants from both samples were then collected by pipetting, diluted 2000‐fold prior to UV–vis analysis, and the absorbance at 554 nm was measured. To examine the release kinetics of rhodamine B depending on light intensity, hydrogels were irradiated with visible light at three different powers (low, medium, and high). Supernatants were collected every 20 min for up to 240 min, and the absorbance at 554 nm was measured for each sample.

#### 4.5.3 Contact Angle Analysis of Hydrogel during Irradiation

For contact angle analysis, 10 µL of hydrogel was formed on a gold wafer. 1 µL of 3 M Fe(III)–citrate solution was dropped onto the wafer, followed by 9 µL of 5% (w/w) CMC–Cat solution. The wafer was then wrapped in aluminum foil and incubated for 1 h under humid conditions to allow Fe(III)–Cat crosslinking. Subsequently, visible light was applied to induce the degradation, and changes in the contact angle were monitored over time during the degradation process.

### 4.6 Characterization

UV–vis absorption spectra were obtained from 200 nm to 800 nm using a UV–vis spectrophotometer (UV‐2600, Shimadzu) with a 2 nm slit width, following solvent baseline correction. For the release study of rhodamine B, each variable was evaluated based on the average results from three independent samples. Analyses were done by using UV–vis spectrophotometer at Total‐Period Analysis Center for Ulsan Chemical Industry of Korea Basic Science Institute (KBSI). The measurement of contact angle was evaluated with SmartDrop (FEMTO‐BIOMED) and averaged based on three different samples for each variable. Rheological measurements of the hydrogel before and after irradiation were performed using Discovery HR 20 (TA Instruments) equipped with a 40 mm diameter plate. The plate and stage temperatures were maintained at 25°C throughout the measurements. Amplitude sweep tests were performed at an angular frequency of 10 rad/s over a strain range of 0.1%–1000%, and flow sweep tests were carried out over a shear rate range of 0.1–100 s^−1^. The visible light source (A160WE, Kessil) used for photoreduction allowed manual adjustment of the output power, and each setting was measured using a digital optical power and energy meter (PM100D, Thorlabs).

## Conflicts of Interest

The author declares no conflicts of interest.

## Supporting information




**Supporting File 1**: asia70725‐sup‐0001‐SuppMat.pdf.

**Supporting File 2**: asia70725‐sup‐0002‐MovieS1.mp4.

## Data Availability

The data that supports the findings of this study are available in the supplementary material of this article.
