# Supplementary material for: Photoreductive Degradation of Fe(III)–Catechol Crosslinked Hydrogels under Visible Light
Source: Chem Asian J. 2026 Apr 2;21(7):e70725. doi: 10.1002/asia.70725 (PMC13047248; doi:10.1002/asia.70725)
Supplement: Supplementary file 1 — Supporting File 1: asia70725‐sup‐0001‐SuppMat.pdf. [file ASIA-21-e70725-s001.pdf]

SUPPLEMENTARY INFORMATION

Photoreductive Degradation of Fe(III)–Catechol Crosslinked Hydrogels under Visible Light

Ukhyeon Kim[a] and Beom Jin Kim*[a]

[a] Department of Chemistry, University of Ulsan, Ulsan 44776, Republic of Korea

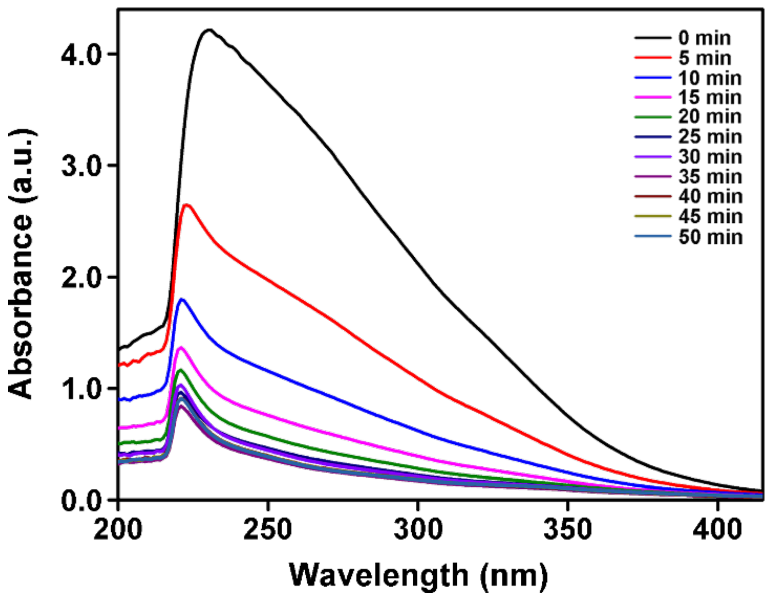

Figure S1. UV–Vis absorption spectra of Fe(III)−citrate complexes, showing a characteristic shoulder peak near 230 nm derived from LMCT transitions.

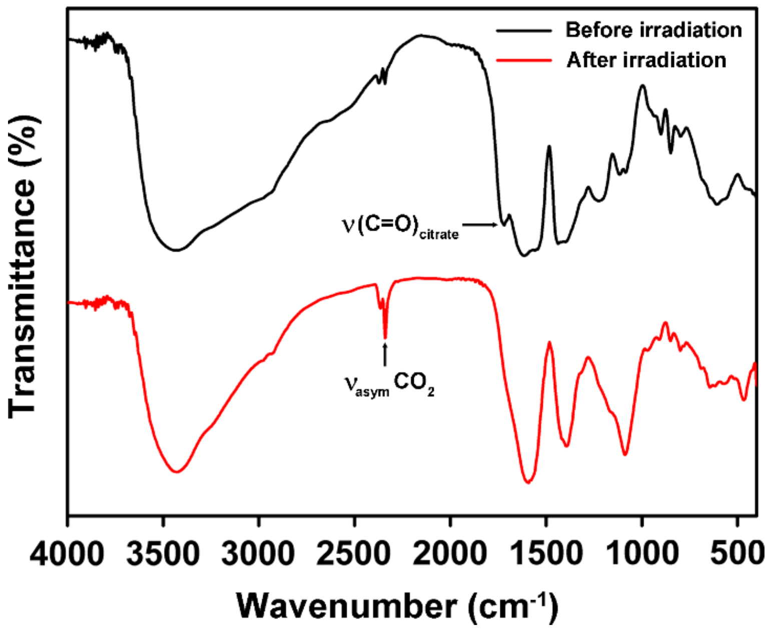

Figure S2. FT-IR spectra of the Fe(III)–citrate complex before and after visible-light irradiation.

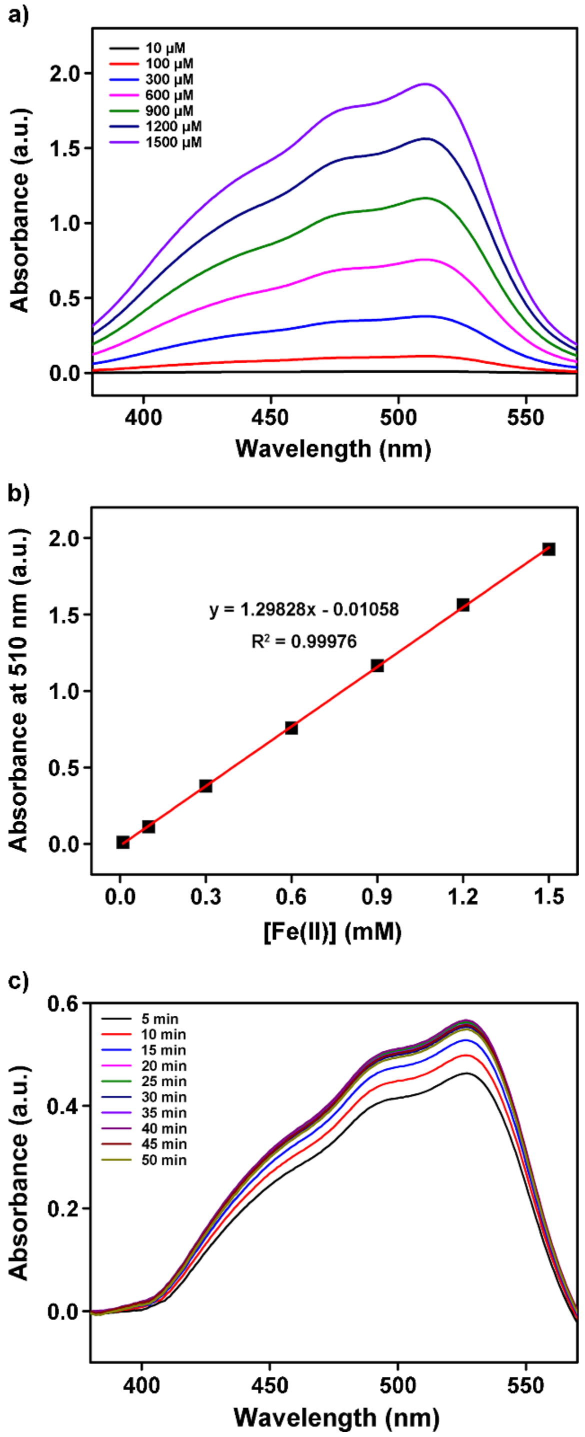

Figure S3. a) UV–Vis absorption spectra of Fe(II)−phen complexes prepared from aqueous Fe(II) solutions of known concentrations, showing a characteristic peak at 510 nm. b) Standard calibration curve of absorbance at 510 nm plotted against Fe(II) concentration. c) Time-dependent UV–Vis absorption spectra of the Fe(II)–phen complex formed during visible-light-induced disassembly of the Fe(III)–Cat crosslinked hydrogel.

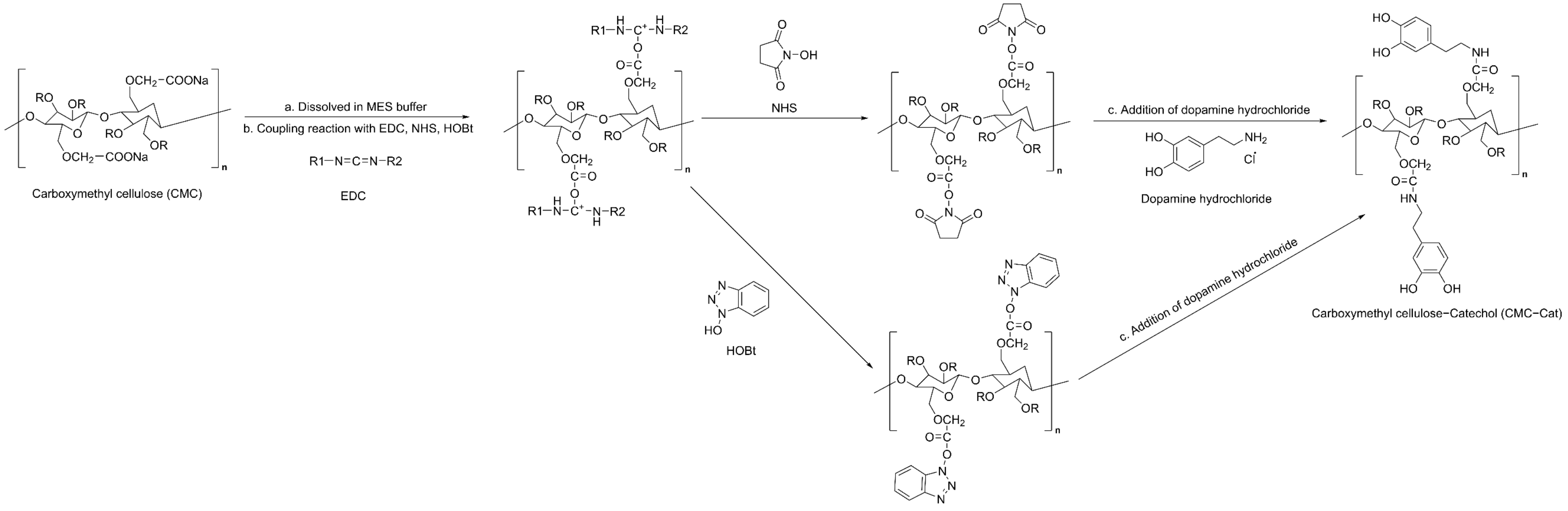

Figure S4. Synthetic scheme for CMC−Cat.

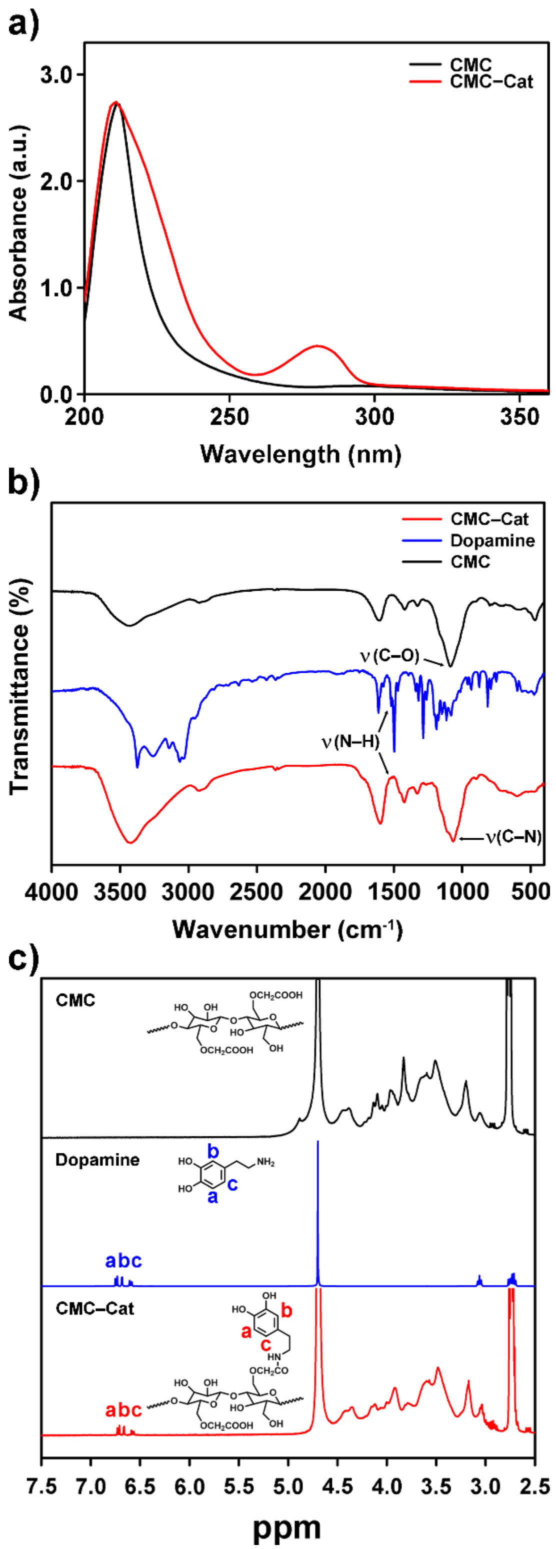

Figure S5. a) UV–Vis absorption spectra of CMC and CMC−Cat, showing a characteristic absorption band near 275 nm for CMC−Cat. b) FT-IR spectra and c) ^{1}H NMR of CMC, dopamine, and CMC–Cat.

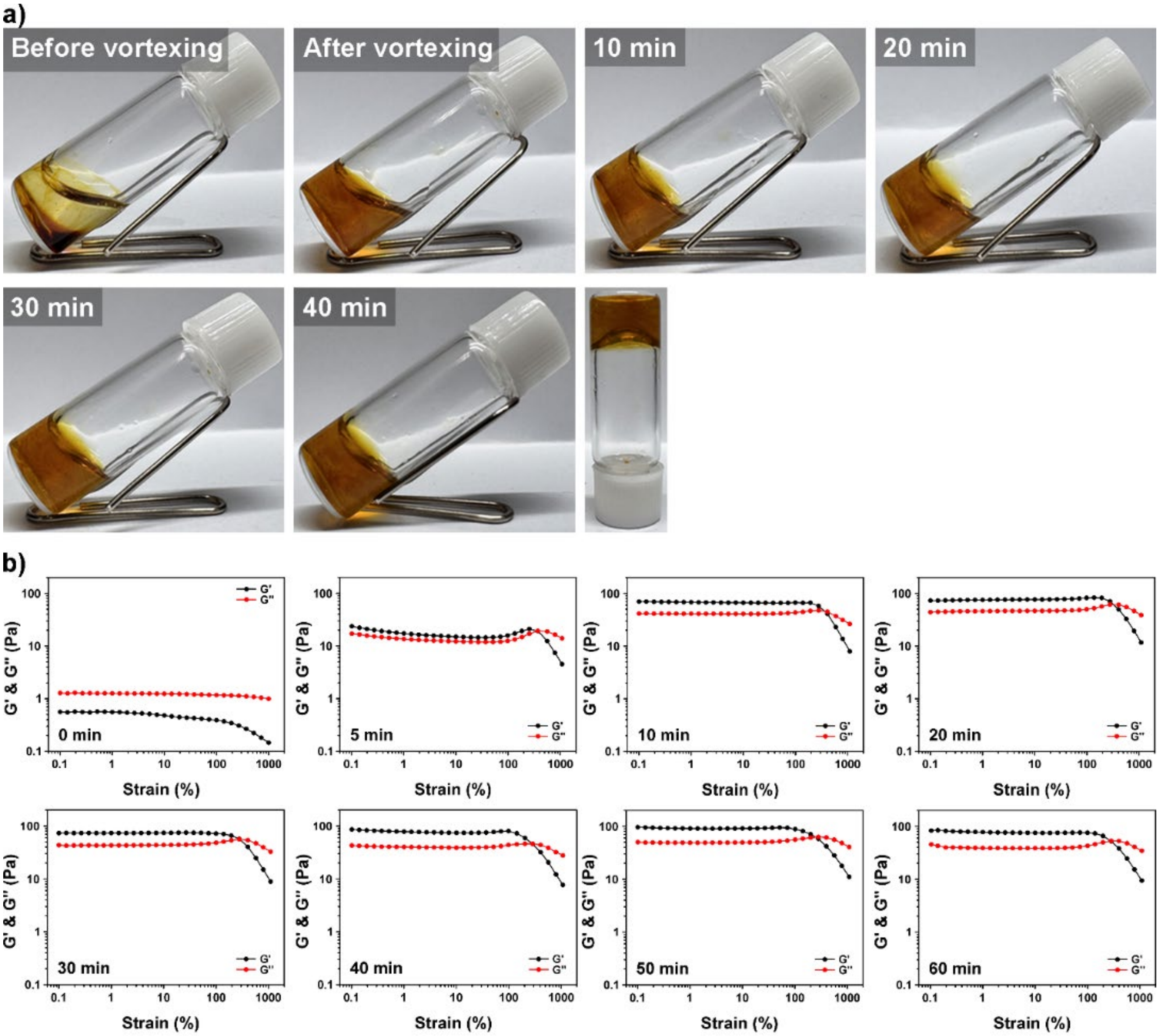

Figure S6. a) Time-dependent gelation process of CMC−Cat with Fe(III)−citrate, showing complete hydrogel formation after 40 min. b) Strain sweep rheological measurements of the CMC–Cat hydrogel at different time points after mixing with Fe(III)–citrate.

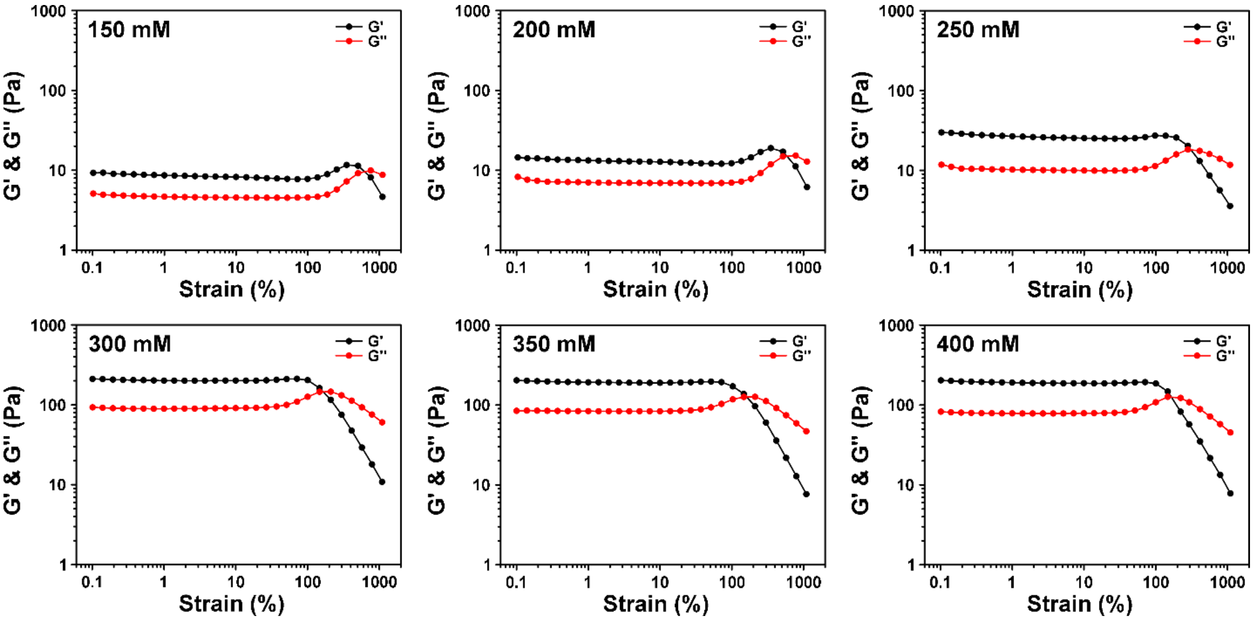

Figure S7. Strain sweep rheological measurements of CMC–Cat hydrogels prepared with different Fe(III)–citrate concentrations. The CMC–Cat concentration was fixed at 4.5% (w/w).

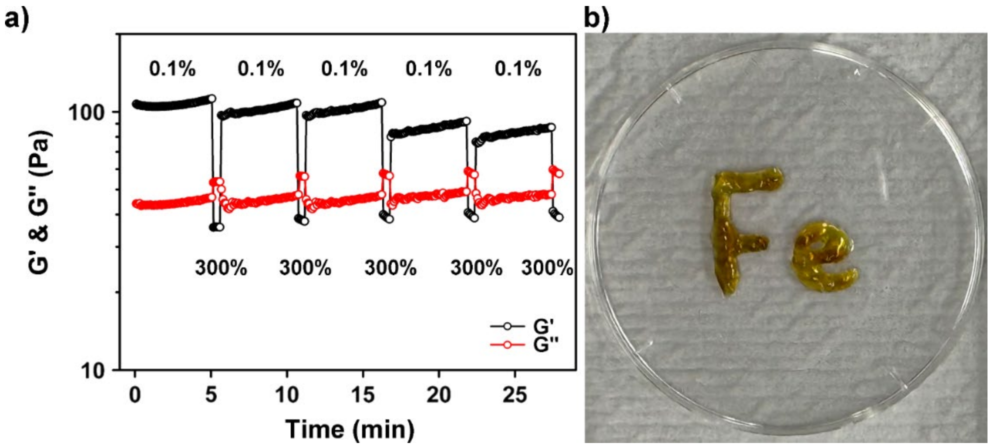

Figure S8. a) Alternating strain sweep rheological measurement showing reversible transitions between elastic-dominant (G' > G'') and viscous-dominant (G'' > G') states under cyclic low (0.1%) and high (300%) strain. b) Photograph demonstrating extrusion of the hydrogel through a syringe.

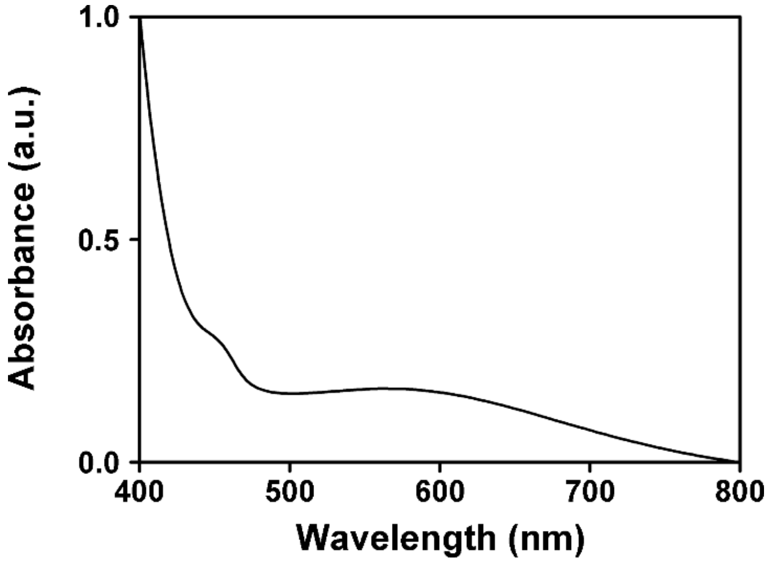

Figure S9. UV–Vis absorption spectrum of Fe(III)–Cat crosslinked hydrogel.

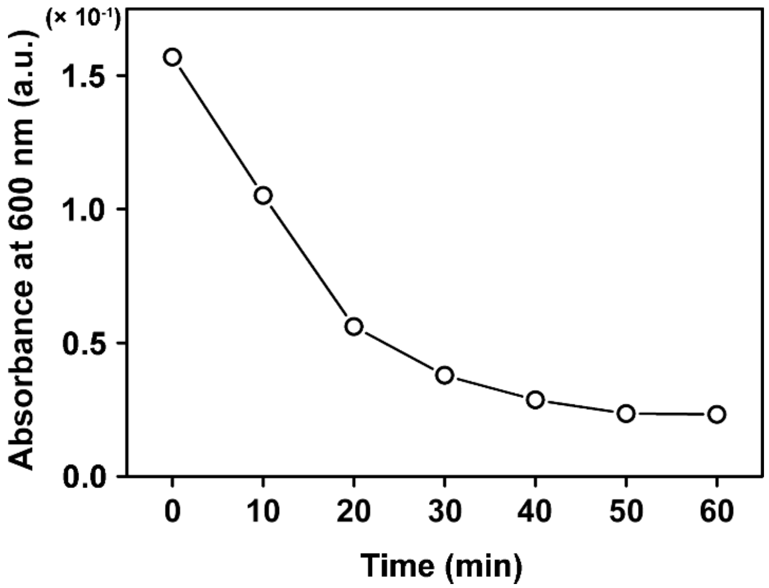

Figure S10. Time-dependent decrease in the LMCT absorption of the Fe(III)–Cat crosslinked hydrogel monitored at 600 nm during visible-light irradiation.

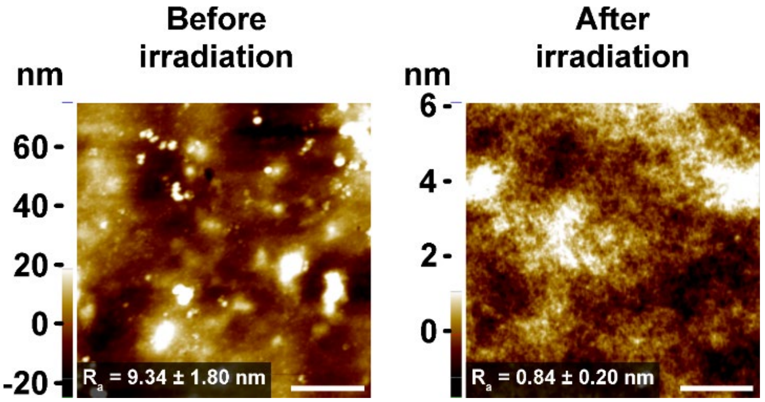

Figure S11. AFM height images of the hydrogel before and after visible-light irradiation. Scale bars: 2 μm.

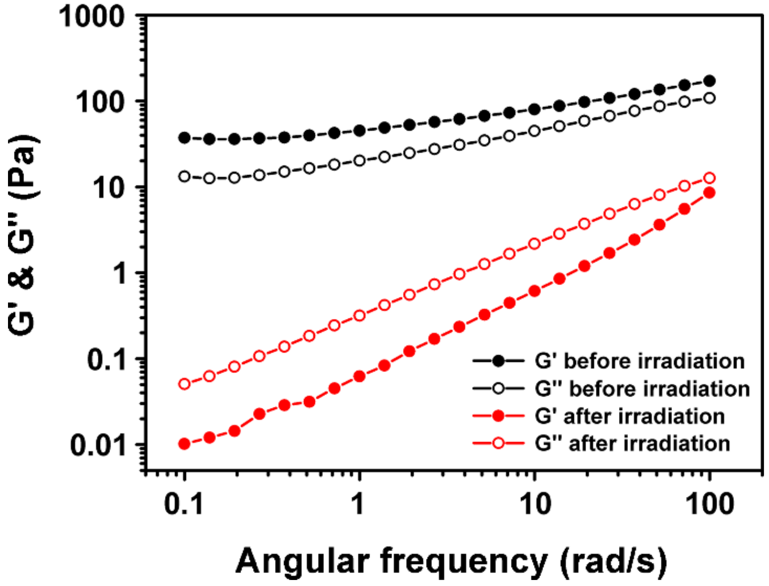

Figure S12. Frequency sweep rheological profiles showing the G' and G'' of the Fe(III)–Cat crosslinked hydrogel before and after visible-light irradiation.

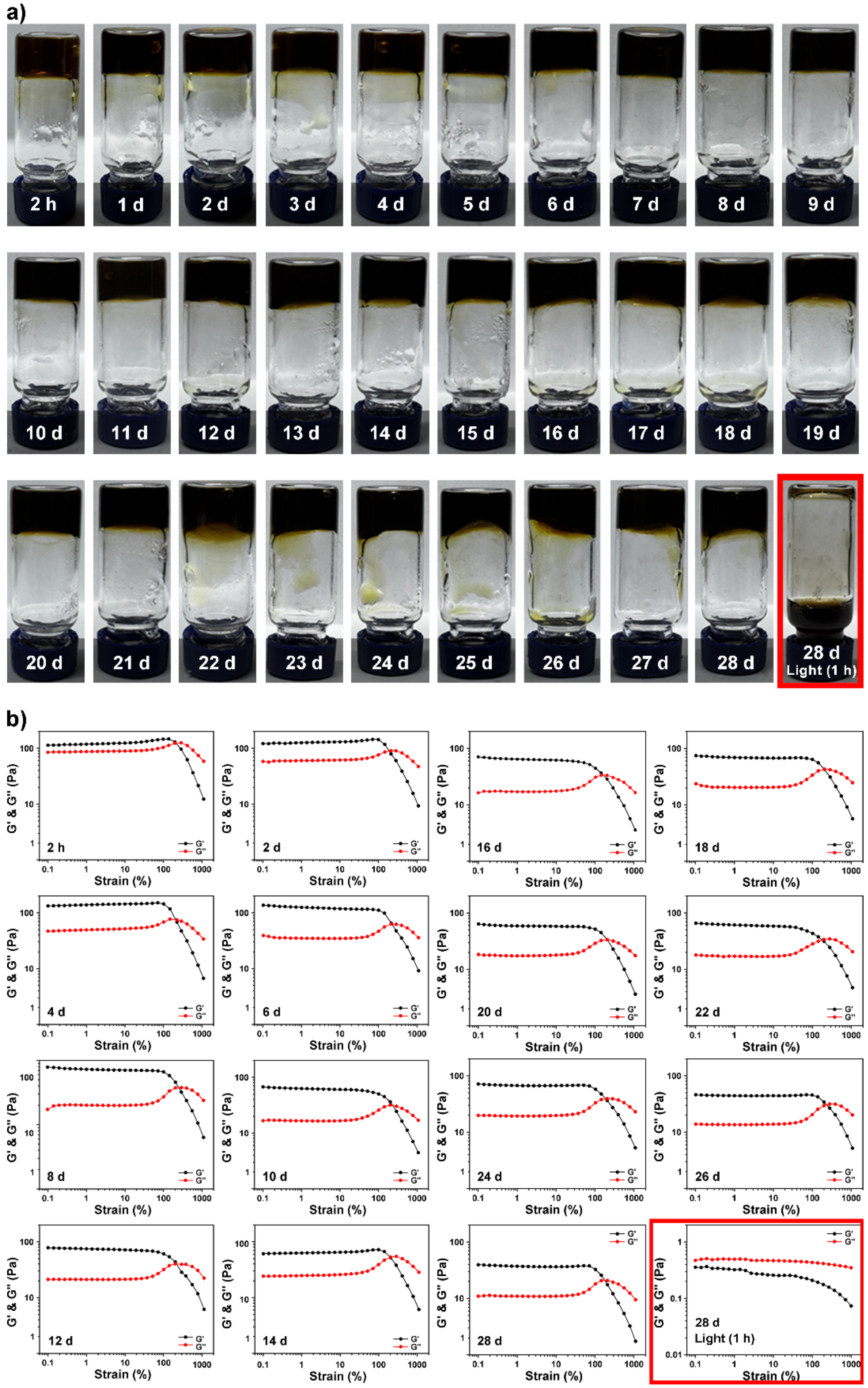

Figure S13. a) Macroscopic images of the CMC–Cat hydrogel maintained at 37 ºC under dark conditions for up to 28 d and after visible-light irradiation (1h). b) Strain sweep rheological measurements of hydrogels incubated at 37 ºC for different time periods.

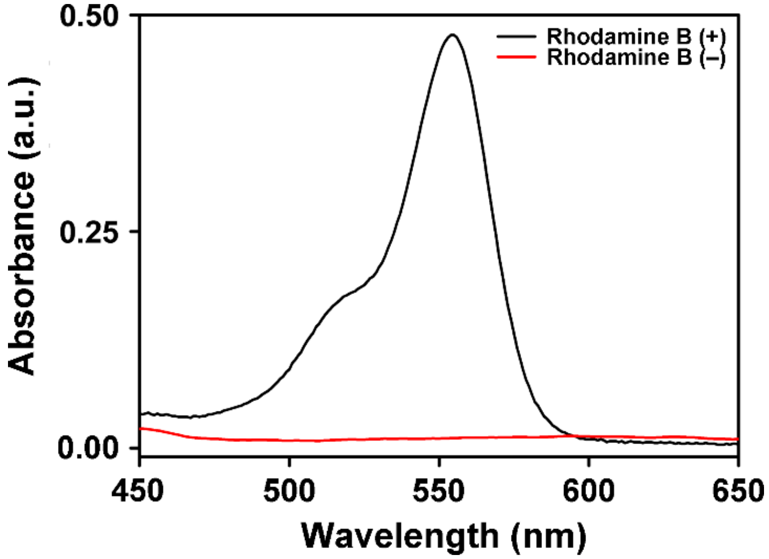

Figure S14. UV–Vis absorption spectra of degraded hydrogels after visible-light irradiation, prepared with (+) and without (−) rhodamine B.

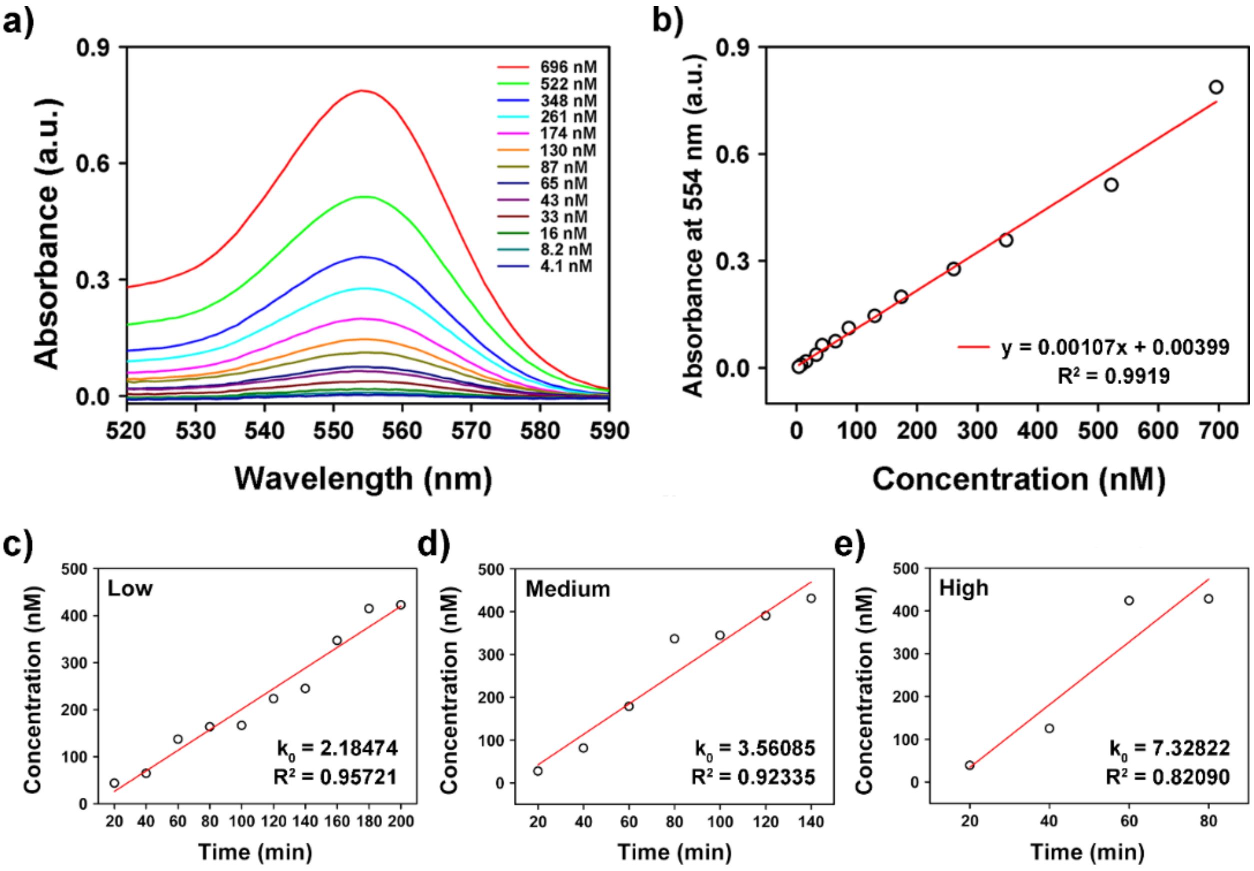

Figure S15. a) UV–Vis absorption spectra of rhodamine B at different concentrations. b) Calibration curve of rhodamine B based on the absorbance at 554 nm as a function of concentration. c-e) Zero-order kinetic plots of rhodamine B release from the Fe(III)–Cat crosslinked hydrogel under visible-light irradiation at c) low, d) medium, and e) high light intensity. Linear fitting of the concentration–time data was used to determine the apparent zero-order rate constants (k_0).

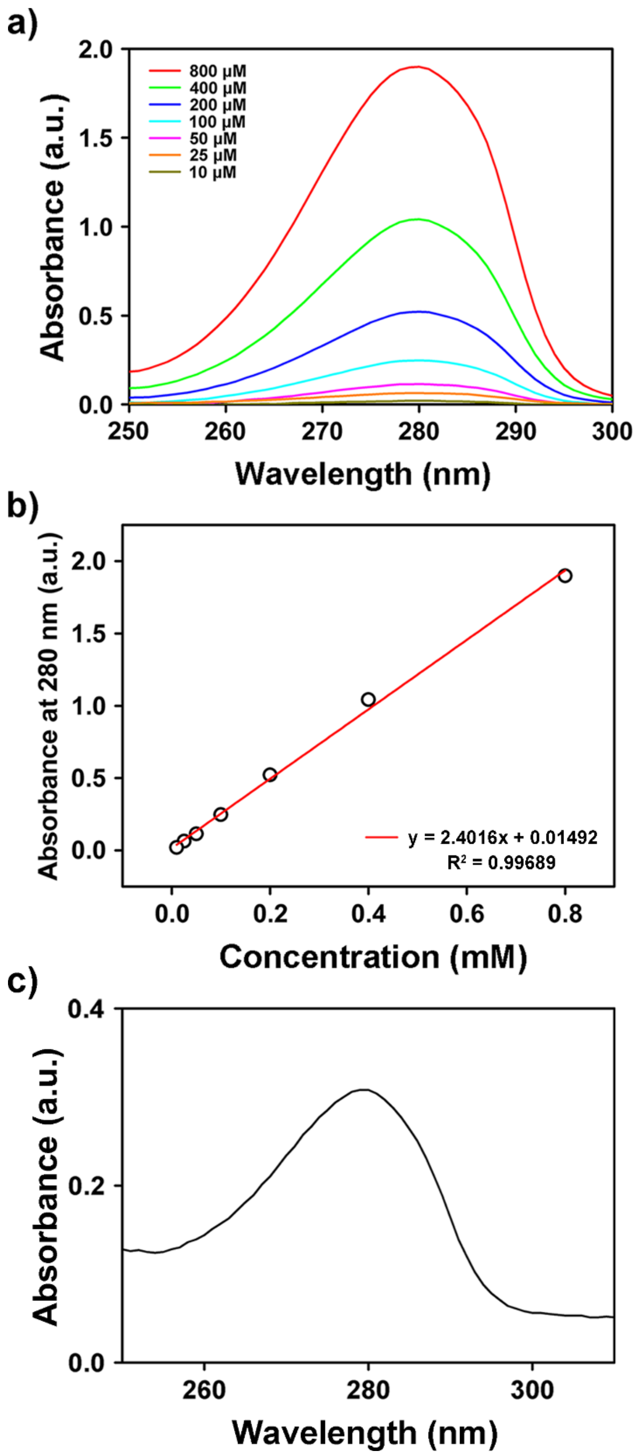

Figure S16. a) UV–Vis absorption spectra of dopamine aqueous solutions at different concentrations measured for calibration. b) Calibration curve obtained from the absorbance of dopamine at 280 nm as a function of concentration, used to quantify the amount of Cat conjugated to CMC for determining the degree of Cat substitution. c) UV–Vis absorption spectra of CMC–Cat.

	Low	Medium	High
**Light power (mW)**	56.5	85.0	111.0
**Light intensity (mW/cm^2)**	71.9	108.2	141.3

Table S1. Light power and corresponding light intensity values for low, medium, and high irradiation conditions.
